# Amphiphilic Polymer Co-Networks

**DOI:** 10.3390/gels6020018

**Published:** 2020-06-10

**Authors:** David Díaz Díaz

**Affiliations:** 1 Departamento de Química Orgánica, Universidad de La Laguna, Avda. Astrofísico Francisco Sánchez, 38206 La Laguna, Tenerife, Spain; ddiazdiaz@ull.edu.es or david.diaz@ur.de; Tel.: +34-922-318584; 2 Instituto Universitario de Bio-Orgánica Antonio González, Universidad de La Laguna, Avda. Astrofísico Francisco Sánchez 2, 38206 La Laguna, Tenerife, Spain; 3 Institute of Organic Chemistry, University of Regensburg, Universitaetsstr. 31, 93040 Regensburg, Germany

**Keywords:** polymeric hydrogels, amphiphilic polymer co-networks, self-organization

## Abstract

*Amphiphilic Polymer Co-networks: Synthesis, Properties, Modelling and Applications* is a new and very interesting book published by the Royal Society of Chemistry and edited by Prof. Costas S. Patrickios (University of Cyprus). Herein, a brief review of the most important features of the book and its contents is provided from a personal perspective.

## 1. Introduction 

Polymeric hydrogels are crosslinked hydrophilic polymers which are capable of absorbing water, having the characteristics of both a solid and a fluid. Their higher water content makes hydrogels more fluid and transparent but also more fragile. Polymeric materials consisting of crosslinked hydrophilic and *hydrophobic* homopolymers, or crosslinked *amphiphilic* block or graft copolymers, are amphiphilic gels or amphiphilic polymer co-networks (APCN). The presence in these materials of the hydrophobic segments, in addition to the hydrophilic ones, allows only limited water absorption (i.e., reduced swelling), rendering them less fluid-like and mechanically stronger than classical hydrogels. Provided that the hydrophobic components come in segments of several consecutive hydrophobic units, these hydrophobic segments aggregate and the APCNs undergo self-organization in water, a process analogous to surfactant aqueous micellization. Upon APCN deformation, these hydrophobic pockets dissipate mechanical energy, rendering these materials stronger. The hydrophobic pockets can also solubilize hydrophobic solutes, such as drugs to be subsequently delivered, or sequester oils or other hydrophobic pollutants for environmental remediation. 

## 2. Book Review

**Figure 1:**
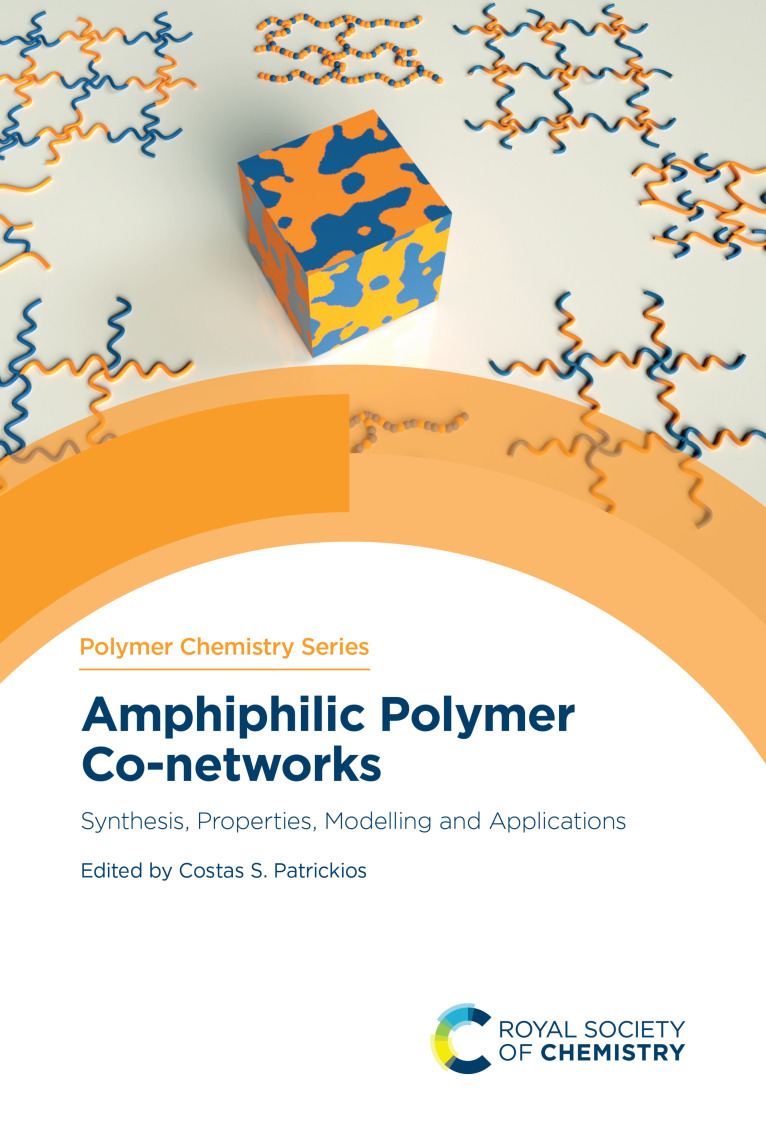
Book Cover, © Royal Society of Chemistry 2020. Other key information: hardcover, 363 pages, £179.00—ISBN 978-1-78801-370-3.

This book [1] (Figure 1) is about APCNs, filling a gap in the literature where one can find several books on related subjects, such as hydrogels, polymer networks, star and hyperbranched polymers, dendrimers, block and graft copolymers, but not on APCNs. This is rather peculiar given the fact that it has now been more than 30 years since the birth of this field, the increasing number of research groups doing research in this field, the widespread importance of silicone hydrogel contact lenses representing a very successful commercial application of APCNs, and the many other APCN applications under development. 

The book’s 15 chapters are written by experts in the field, coming from four continents and 12 countries, and are organized into four sections that include synthesis, properties, modeling and applications. The chapters describe and discuss new developments in the field, while the history of APCNs since their origin in 1988 is summarized in the Foreword by Prof. J. P. Kennedy of the University of Akron and in Chapter 1 by the editor of the book. A brief outlook for the field is provided at the end of Chapter 1. The co-network architectures discussed in this book are mainly derived from hydrophobic macromolecular crosslinkers which represent the classical approach; however, more modern designs as well as the use of more efficient coupling chemistries are also presented. Among different APCNs, the book emphasizes those based on the combination of hydrophilic components such as poly(*N*-vinylimidazole), poly(ethylene glycol), poly[(2-dimethylamino)ethyl methacrylate], polysaccharides or poly(ethylene glycol)s, among others, and hydrophobic domains derived, for instance, from poly(tetrahydrofuran), polyisobutylene, poly(ε-caprolactone), four-armed star poly(ethyl glycidyl ether)s and poly(dimethylsiloxane)-based crosslinkers. Important properties discussed throughout different chapters include aqueous swelling, thermophysical and mechanical properties, self-assembly mechanism, morphological features, external stimuli responsiveness, (e.g., thermal, pH, light, salt or electrical actuation), degradation and protein adsorption. In addition, useful comparisons between the properties of these materials and those of common polymeric hydrogels are provided for important examples. Applications described in the book chapters include, for instance, the use of APCNs in soft contact lenses, drug delivery, tissue engineering, heterogeneous biocatalysis (including enantioselective catalysis), and for the fabrication of membranes of controllable permeability and antibacterial hybrid materials. Furthermore, an important theory chapter on the simulation of the self-assembly of APCNs, focusing on microphase separation and order–disorder transitions, is also included.

## 3. About the Book Editor

Professor Costas S. Patrickios obtained his PhD in 1993 at the Massachusetts Institute of Technology (MIT) in Cambridge, Massachusetts in the USA. Between 1994 and 1996, Dr. Patrickios worked as a post-doctoral researcher at the University of Sussex in the UK. Then, he served as a Lecturer at the University of Manchester Institute of Science and Technology (UMIST) (1996–1997), also in the UK. In 1998, he joined the Department of Natural Sciences (now Department of Chemistry) at the University of Cyprus (Republic of Cyprus), where he has remained since then. His main research has been focused on the synthesis and characterization of amphiphilic polymer co-networks based on interconnected amphiphilic block copolymers prepared using the living/controlled polymerization techniques group transfer polymerization (GTP), atom transfer radical polymerization (ATRP) and reversible addition-fragmentation chain transfer (RAFT) polymerization. He is currently the Chair of the Polymer Networks Group (PNG), an international organization promoting research on polymer networks and gels.

## 4. Conclusions

In conclusion, this is a timely and decisively important book with well-articulated content, which invites the hydrogel research community to consider adding a hydrophobic block/segment to their materials in order to convert them into amphiphilic gels and examine whether the resulting properties are improved for the particular application compared to those of the original hydrogels. The book’s style and its content diversity make it appropriate for graduate students and researchers interested in hydrogels, polymer chemistry, polymer networks, self-assembly and nanomaterials. Certainly, this is a must-read book for all those who are passionate about engineering new functional hydrogels that can contribute to solving real-world challenges.
